# Scientific Items

**Published:** 1880-10-20

**Authors:** 


### SCIENTIFIC ITEMS.

**Rather Old Butter.**—At a late meeting of the Society of Public Analysis, in London, a paper by Prof. Church and Mr. Wigner, "on two ancient samples of butter" was read, of which the Chemical News gives the following abstract:

The first was a sample of Irish bog butter, and its probable age was judged to be about one thousand years. The sample contains nearly 4 per cent of curd, which consisted partly of vegetable matter derived from the bog, but contained quite enough animal matter to prove that the butter had been originally made from animal milk and was not a mere artificial fat. Its fatty character had, however, been entirely changed, and the glycerides of which the fat had originally consisted had been decomposed so as to leave simply a mixture of the fatty acids which constitute the acid portion of animal fats. The butter had, in fact, become changed into a substance closely resembling in character and composition the substance of which good composite candles are composed. The result is singular, as showing that length of time combined with exposure to moisture will effect the decomposition which the manufacturer of stearine has to effect by the agency of heat and acids.

The other and older sample of butter had been taken from an alabaster vase in an Egyptian tomb; it had evidently been melted and poured into the vase, and carefully sealed over. This sample was probably about 2500 years old, but the preservation had been so perfect that it was only slightly rancid, and had fully retained the chemical properties of genuine butter, the fat not having been decomposed to any sensible extent. This sample possessed a decided taste and smell of butter, while the sample from the bog was cheesy rather than buttery in smell.—*Boston Journal of Chemistry.*

**An Electric Alarm for Firemen.**—Superintendent Willis, of the Salford, (Eng.) Fire Brigade, has constructed a unique fire alarm. On a notice being received the man on duty, while receiving particulars from the informant, presses a single knob in the room, which causes the large alarm bell to be rung, arouses each man of the permanent staff, who live in or near the fire station, opens the doors of the stable and engine-house, turns on the different jets of gas, and finally runs the engine out into the yard. It is calculated that in case of fire fifty seconds will suffice in which to do work that hitherto has taken three minutes—*Boston Journal of Chemistry.*

**Electricity in the Human Body.**—Most people are familiar with the "sparks" which may be produced under certain conditions by stroking the fur of a cat; and travelers in Canada and other cold, dry countries have witnessed the still more remarkable phenomenon of the human body being turned into a conductor of electricity, and the possibility of lighting the gas by merely placing one's finger—given the necessary conditions of electrical excitement—near the gas jet, without any other agency. Mr. A. W. Mitchison, the African traveler, who is engaged in writing a narrative of his exploring expeditions in West Central Africa, gives some still more startling facts. He states that one evening, when striking an African native, in a moment of anger, with a cowhide whip, he was astonished to see sparks produced, and still more surprised to find that the natives themselves were quite accustomed to the phenomenon. He subsequently found that a very light touch, repeated several times, under certain conditions of bodily excitement, and in certain states of the atmosphere, would produce a succession of sparks from the bodies of native men as well as native cattle. A lazy nigger, it seems, yielded none of these signs of electricity. We are not aware that these facts have been recorded by other travelers, but they certainly deserve thorough sifting by competent observers.—*Lancet.*

**Animal Electricity.**—According to La Nature, Mr. Marey has arrived at the conclusion that "the electricity in the apparatus of the torpedo and muscular work are produced under similar physiological conditions."—*Ib.*

**Atoms.**—Great Britain manufactured last year 1,545,500,000 gallons of beer, and the United States 336,300,000 gallons; the account for Germany cannot be totalled by any known mathematician.

AN important discovery has just been made at Bath, England, in the shape of a perfect Roman bath some 80 feet long and 40 feet wide, the whole of the interior being lined with lead three quarters of an inch in thickness; the bath is some 50 feet below the level of the present surface.

TELEPHONIC effects result from the shock of magnetic bodies, according to Mr. Ader, who says that any mechanical action that disturbs the molecular condition of a magnet core develops, when the core suddenly regains its equilibrium, an electric current capable of affecting the telephone.

THE reports of the British inspectors of mines for 1879 show that 1037 lives were lost in the mines of the United Kingdom in that year, against 453 in 1878; the number of persons employed in the mines was 522,807.

IT IS said that more people lost their lives in the States by the burning of hotels in 1879, than by the accidents of travel on railroads and steamboats combined.

IT IS reported that 320,000 holes were bored in the execution of the St. Gothard Tunnel, 980,000 lbs. of dynamite consumed, and 1,650,000 drills worn out.

THE Volksgarten, a large musical promenade in Vienna, is to be illuminated by means of thirty Jablochkoff candles.

A YANKEE has invented a machine for making the sides of a wooden pail in one piece.

A NEW battery is being produced, having one of its elements composed of sheet iron less than the ten thousandth of an inch in thickness.

